# An overview and visual analysis of research on government regulation in healthcare

**DOI:** 10.3389/fpubh.2023.1272572

**Published:** 2023-11-13

**Authors:** Min Qi, Jianming Ren

**Affiliations:** School of Public Administration, Beihang University, Beijing, China

**Keywords:** government regulation, healthcare, VOSviewer, visual analysis, bibliometric analysis, COVID-19

## Abstract

**Objective:**

During the period of COVID-19, government regulation (GR) played an important role in healthcare. This study examines the current research situation of GR in healthcare, discusses the research hotspots, the most productive authors and countries, and the most common journals, and analyzes the changes in GR in healthcare before and after the outbreak of COVID-19.

**Methods:**

This study followed PRISMA guidelines to collect literature on GR in healthcare. And the VOSviewer software was used to perform a quantitative analysis of these documents to obtain a visual map, including year, country, institution, journal, author, and research topic.

**Results:**

A total of 1,830 papers that involved 976 academic journals, 3,178 institutions, and 133 countries were identified from 1985 to 2023. The United States was the country with the highest production (*n* = 613), followed by the United Kingdom (*n* = 289). The institution with the largest number of publications was the University of London in the UK (*n* = 103); In the author collaboration network, the biggest cluster is Bomhoff M, Bouwman R, Friele R, et al. The top five journals in terms of the number of articles were BMC Health Services Research (*n* = 70), Plos One (*n* = 35), Health Policy (*n* = 33), Social Science & Medicine (*n* = 29), Health Policy and Planning (*n* = 29), and Frontiers in Public Health (*n* = 27). The existing literature mainly focused on “health policy,” “public health,” “China,” “mental health,” “India,” “qualitative research,” “legislation,” and “governance,” et al. Since 2020, research on “COVID-19” has also become a priority in the domain of healthcare.

**Conclusion:**

This study reveals the overall performance of the literature on GR published in healthcare. Healthcare needs GR, especially in response to the COVID-19 epidemic, which has played an irreplaceable role. The outbreak of COVID-19 not only tested the health systems of various countries, but also changed GR in healthcare. With the end of COVID-19, whether these changes will end remains to be further studied.

## Introduction

GR is divided into economic regulation and social regulation. Japanese scholar Masu Uekusa divided social regulation into four categories, including ensuring health and hygiene; ensuring safety; preventing public hazards and protecting the environment; ensuring education, culture, and welfare (1). It can be seen that healthcare regulation is one of the important contents of government social regulation. In most wealthy countries, healthcare is strictly regulated (2). This is because the healthcare sector is a field where diverse interests are intertwined, requiring government involvement to develop appropriate transaction orders. At least since Arrow, government participation in the healthcare industry has been seen as an important measure that may overcome market failures (3). For example, in a model similar to Aklov’s lemon market, Leland showed that minimum quality standards can prevent low-quality suppliers from driving out high-quality suppliers (4). Especially with the development of science and technology, higher requirements have been put forward for the regulation of biotechnology. In addition, HIV, mad cow disease, the threat of global epidemics, new technologies, and the potential of human cloning have further increased the public demand for more regulation (5).

The outbreak of COVID-19 has brought new challenges to global healthcare. Governments around the world have taken various measures to actively respond to COVID-19, such as formulating or adjusting relevant policies or regulations on telemedicine (6, 7). It can be seen that the global epidemic is not only a test of the existing health systems of countries, but also further changes government intervention in healthcare. As a concern for practical issues, what are the changes in academic research on GR? Based on this problem, it is necessary to review and sort out the current literature on GR.

Currently, some researchers reviewed the literature on GR in some parts of healthcare, such as the research on GR of private health insurance (8), and the impact of drug price regulation on access to essential medicines and drug innovation (9). Although these studies have provided some insights, they did not use a set of mathematical and statistical methods to comprehensively and systematically analyze the quantity and quality of publications through bibliometrics. Such an analysis can help researchers understand the current research status and future development trend of GR in the field of healthcare. Therefore, in the context of the end of COVID-19, and to help realize the current global evidence landscape on GR studies, we conducted a new bibliometric analysis of GR research to describe patterns of global cooperation and map trends of GR over the past few years.

The purpose of this study is to: (1) analyze the distribution of publication output, countries, institutions, authors, journals, and keywords of GR in healthcare; (2) identify the cooperation of authors and institutes; (3) and discuss the hot topics of GR research in healthcare, as well as the focus of research in different regions, which will help readers to understand more about GR in healthcare. The following two suggested research questions (RQ) will help achieve the goals of this study.

RQ1: What are the most important topics explored in the academic literature on GR in healthcare?

RQ2: Concerning COVID-19 pandemic, what measurable changes in content has the COVID-19 epidemic brought to the professional literature on GR in healthcare?

## Methods

### Data source

Considering the special requirements of bibliometric software for data, the publications of this study were sourced from the Web of Science Core Collection (hereinafter referred to as WoSCC) database, including “Science Citation Index Expanded,” “Social Sciences Citation Index,” “Arts&Humanities Citation Index,” “Emerging Sources Citation Index,” “Conference Proceedings Citation Index-Science,” and “Conference Proceedings Citation Index-Social Science & Humanities,” performed from 1 January 1985 to 23 July 2023. WoSCC is a globally leading citation database that includes over 21,800 authoritative and highly influential academic journals from around the world, covering fields such as natural sciences, engineering technology, biomedicine, social sciences, art, and humanities, and has high representativeness and authority. The searching terms were as follows: TS = (government regulation OR government supervision) AND TS = (health care OR healthcare OR health-care).

Finally, literature records, including authors, titles, source publications, abstracts, keywords, institutions, affiliations, and addresses, downloaded as “plain text,” formed the local database for subsequent analysis with Excel and VOSviewer.

### Bibliometric analysis and visualization

Bibliometrics, or bibliometric analysis, is a research field of library and information science, which uses quantitative methods to study bibliographic materials (10). The concept was introduced in 1969 by Alan Pritchard (11). As a scientific research method, bibliometric analysis uses existing research as materials to summarize and analyze the current research status of related topics and predict their future research trends. This method helps researchers to have a precise understanding and grasp of a certain topic. With the development of information technology, various software has been developed for bibliometric analysis, such as Vosviewer, Citespace, and other software. The VOSviewer was developed by the Centre for Science and Technology Studies of Leiden University in the Netherlands for bibliometric analysis. The software collects bibliographic data and provides a graphical map of bibliographic coupling, co-authors, co-occurrence of author keywords, and co-citations (10). Among them, the co-occurrence analysis method mainly counts the number of occurrences of a pair of words in the same literature, and measures the near or far relationship between them through the frequency, which can further understand the development of the research in this field and reveal the structure of the research (12). Compared to CiteSpace software, VOSviewer provides better visualization accuracy for high-frequency keyword analysis (13). At present, VOSviewer software is used for bibliometric analysis in many fields, for example, entrepreneurial intention in the field of business (14), earnings management in the field of management (15), and fatigue during pregnancy in the field of public health (16). Therefore, in this study, we used Vosviewer 1.6.16 software to generate visual graphics.

Specifically, this paper used Microsoft Office Excel 2016 to manage the data, and analyze the publication trend with linear regression. Literature data including countries, authors, and keywords were extracted from the WoSCC search results. This data was then entered into the VOSviewer (1.6.16) software.

1,830 articles in CSV format were entered into VOSviewer and a co-occurrence analysis graph was generated, which consists of multiple nodes and lines. The size of nodes in the graph represents the number of keyword occurrences, and the more keywords appear, the larger the node. The thickness of the line represents the strength of the connection, and the thicker the line, the stronger the connection. Nodes can have different colors, and the same color represents the same cluster. Cluster analysis based on a co-occurrence network is the specific application of the cluster method in a co-occurrence network. It is a quantitative processing technology that takes co-occurrence intensity as the basic unit of measurement to classify and aggregate a given keyword co-occurrence set. This technology can aggregate closely related keywords into one cluster (17).

## Results

### Search results

**Figure 1:**
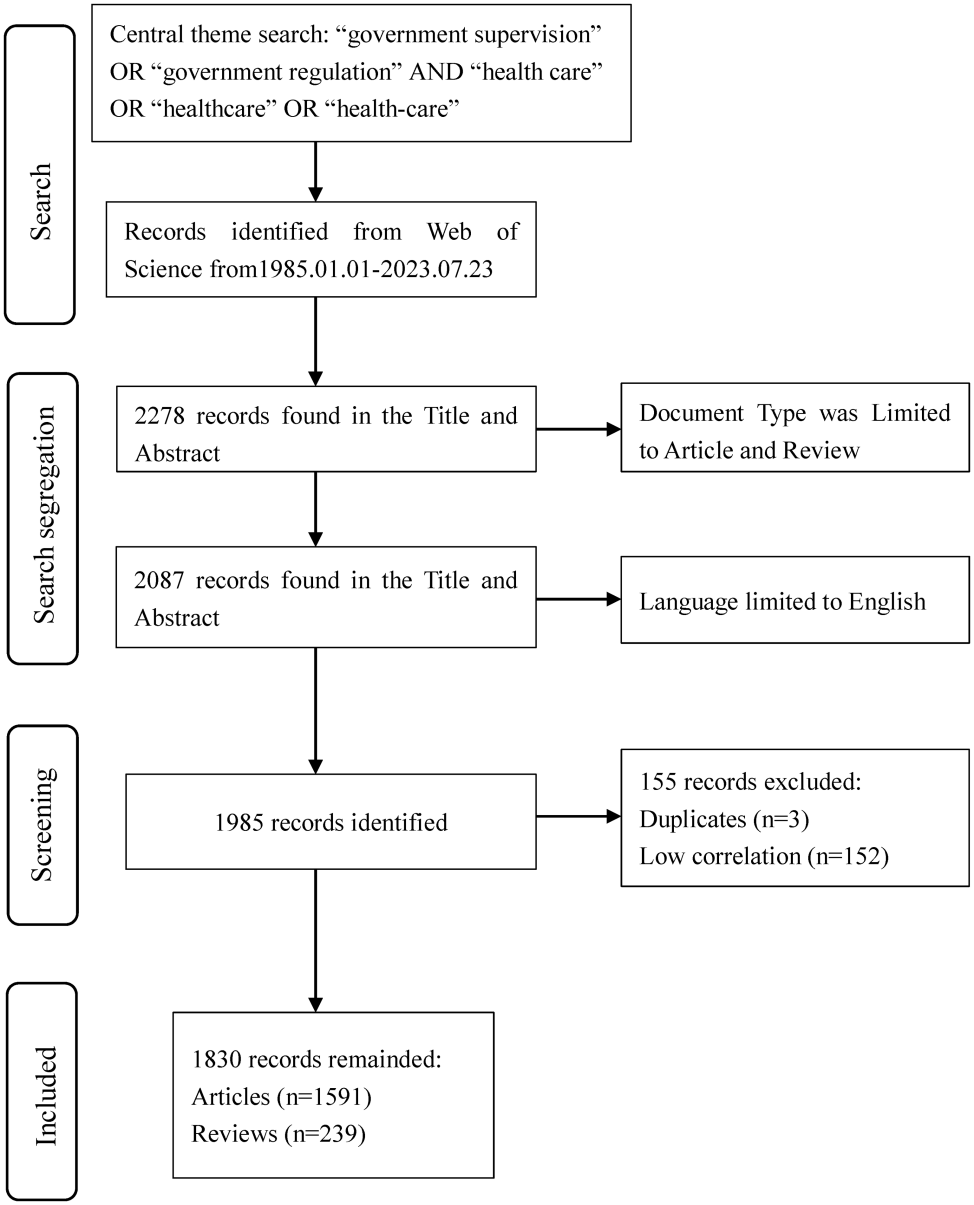
PRISMA flow diagram of data collection based on central search theme.

All documents were uniformly searched and downloaded on 8 October 2023, which helps to avoid data volume biases due to dynamic changes in the database. Through searching in the WoSCC database, a total of 2,278 records were retrieved, and 2,087 literatures were obtained by selecting the literature types as articles and reviews. We limited our search results to papers published only in English and obtained 1,985 papers. Then, 3 duplicates and 152 documents with low relevance to the research topic were deleted. Finally, a total of 1,830 literatures were used for bibliometric analysis (Figure 1), including 239 reviews and 1,591 papers.

### Publication outputs

**Figure 2:**
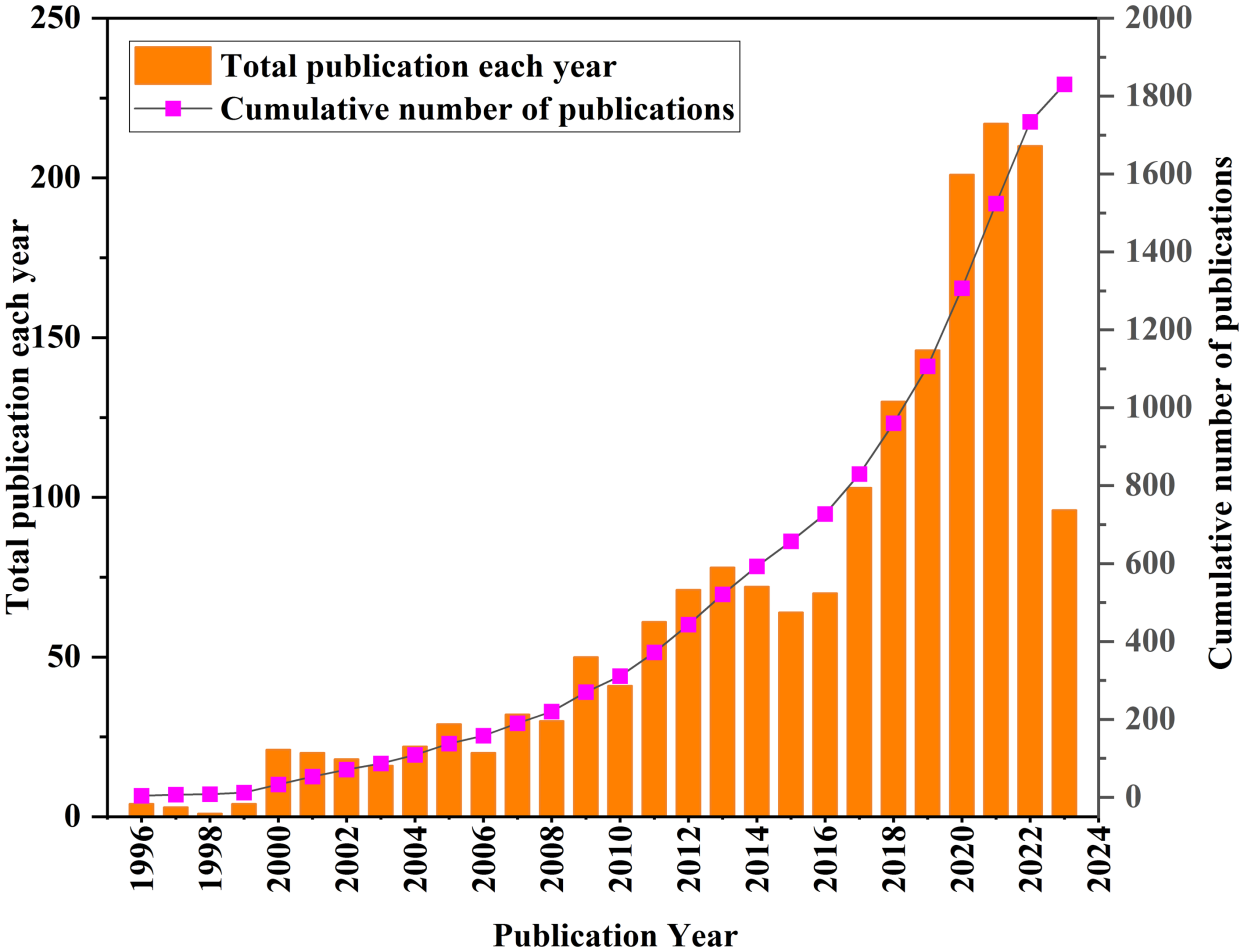
Annual trend chart of publications.

**Figure 3:**
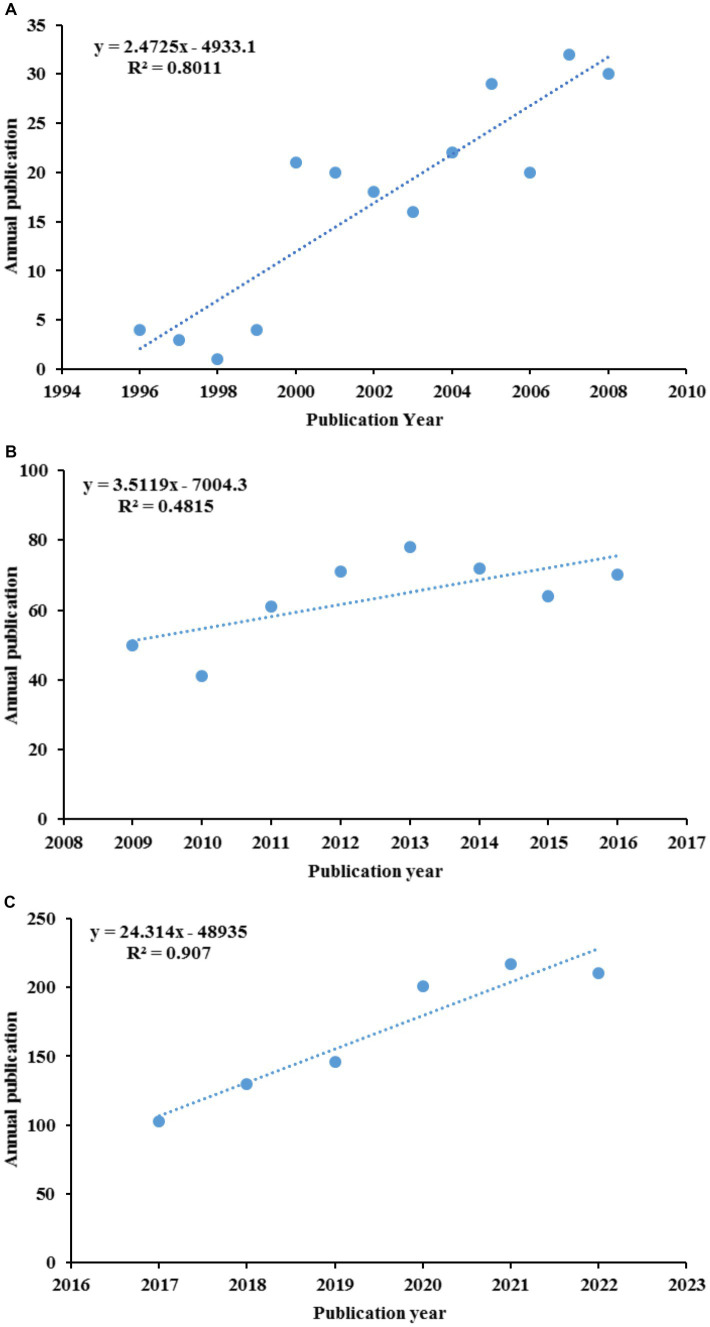
The curve fitting results of annual publications: **(A)** 1996–2008; **(B)** 2009–2016; **(C)** 2017–2022.

The annual growth trends of publications related GR in healthcare are shown in Figure 2. The number of publications has shown an upward trend in fluctuations, with a significant increase in production in the last 6 years (2017–2022, 55%). The earliest published literature was in 1996, with an average annual increase of 21.4 articles from 2018 to 2022. According to the characteristics of the published results, a research period is divided into three stages (1996–2008, 2009–2016, and 2017–2022; Figures 3A–C). In the period 1996–2008, the number of publications was only 220 articles, and the number of publications showed a state of fluctuation. The number of publications from 1996 to 1999 was relatively small, with a small increase to 21 in 2000. Therefore, the research on GR in healthcare was relatively slow in this period; In the period 2009–2016, the number of publications increased to a total of 507; The number of publications in the period from 2017 to 2022 has rapidly increased, with a total of 1,007 articles. In 2020, the growth rate was the highest, reaching 55 articles.

### Regions and institutes

**Figure 4:**
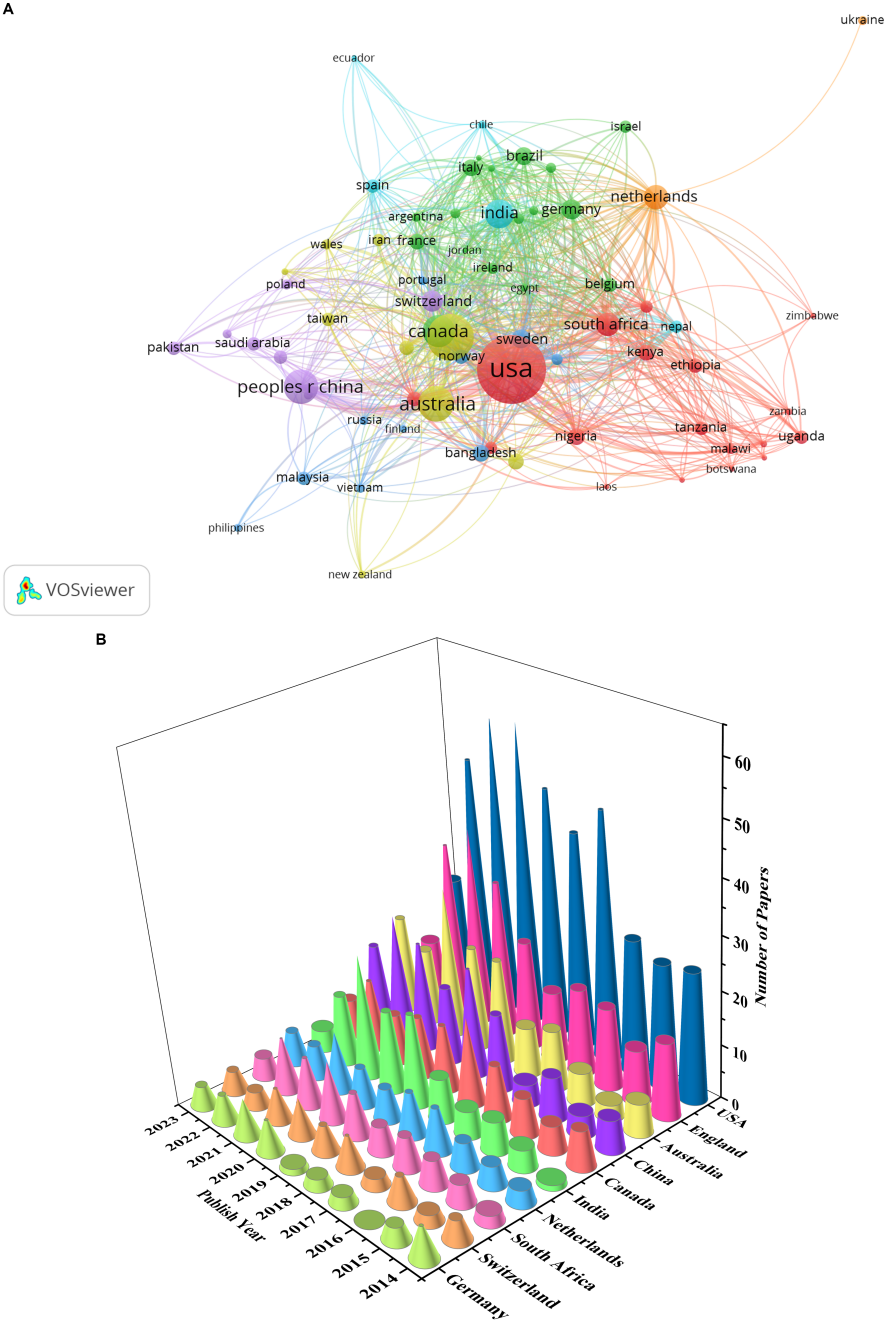
Countries of publications: **(A)** collaboration network of countries; **(B)** the top 10 countries in terms of publication quantity.

A total of 133 countries published articles on GR in healthcare, with 56 countries publishing more than 10 publications (Figure 4A). The top 5 countries ranked by number of publications were United States (613), the United Kingdom (289), Australia (166), Peoples R China (150), and Canada (135). Figure 4B shows the top 10 countries in terms of publication quantity from 2014 to 2023. The cone apex represents the peak of the number of publications. It can be seen that as of July 2023, the peak of the number of publications in the United Kingdom, Australia, India, the Netherlands and Germany is in 2021, while the peak of the number of publications in the United States, South Africa and, Switzerland is in 2020. In addition, the peak number of publications in China is in 2022, while Canada’s is in 2018.

**Figure 5:**
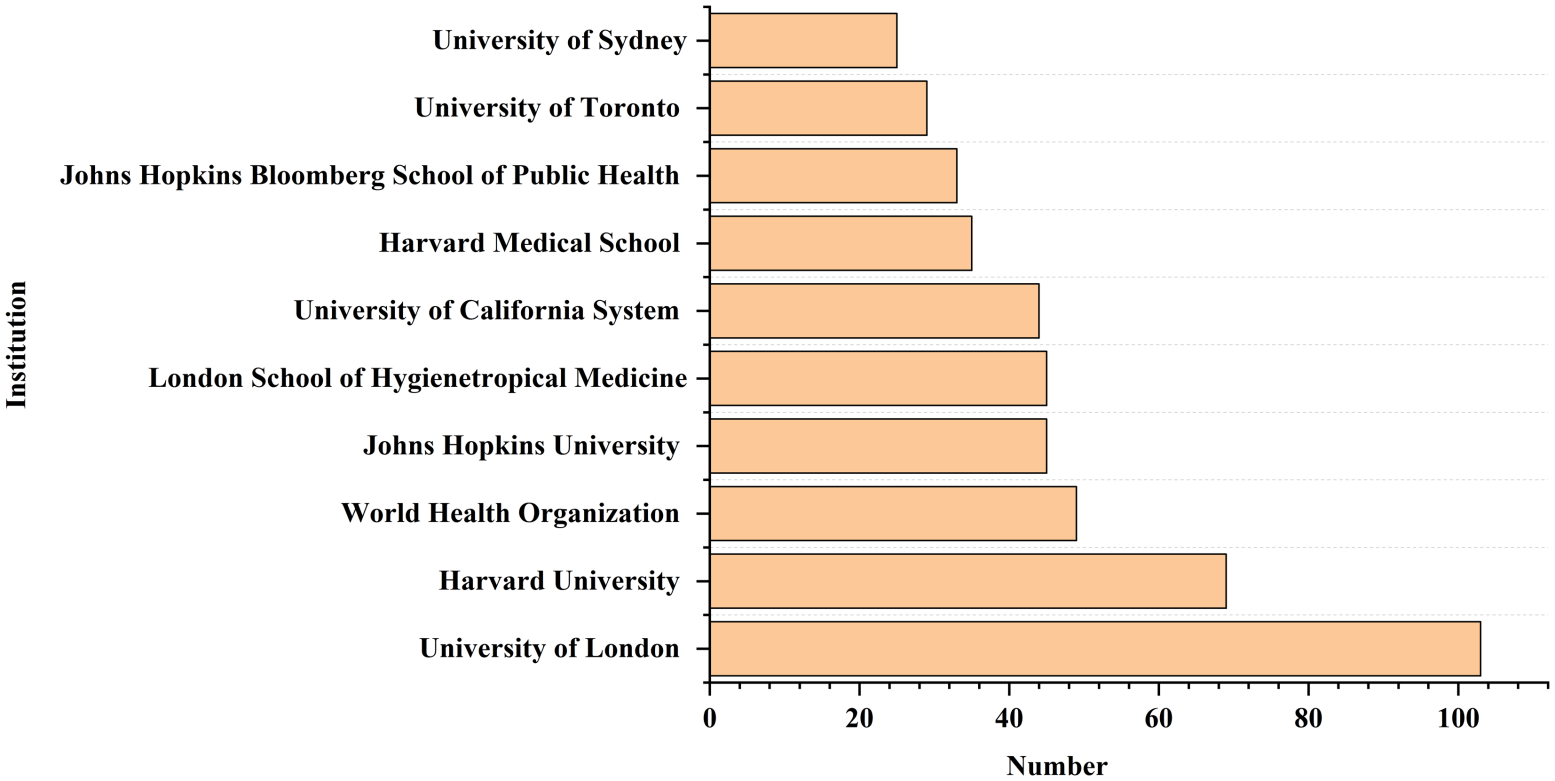
The top 10 institutions in terms of publication quantity.

A total of 3,178 institutions published articles related to GR, out of which 88 institutions published more than 10 papers (Figure 5). The top leading institution by publication count was University of London (*n* = 103) in the UK, mainly published by the London School Of Hygiene And Tropical Medicine Faculty Of PublicHealth And Policy and University College London School Of Life And Medical Sciences. Since 2000, University of London, Johns Hopkins University, and Johns Hopkins University published 69, 45, and 45 articles, respectively. Besides, Harvard Medical School and University of Toronto published 35 and 29 articles, respectively, since 2006. Finally, World Health Organization, University of California System, Johns Hopkins Bloomberg School of Public Health, and University of Sydney each published 49,44,33 and 25 articles up to 2023.

### Authors’ analysis

A total of 7,892 authors published articles related to GR. A total of 8 authors with four or more publications were from India, the United Kingdom, South Africa, the United States and the Netherlands (Table 1). As shown in Table 1, the author with the highest number of publications is Menon, P. from India, who works at the International Food Policy Research Institute (IFPRI), with an h-index of 39. Author Manchikanti, L. from USA, ranked number 1 according to the study with 310 publications since 2007, 78 h-index, and 18,434 citations.

**Table 1:** The author with 4 or more publications.

No.	Author	Country	Number of publications	Total citation	H-index
1	Menon P	India	5	4,838	39
2	Goodman C	England	4	4,994	41
3	George A	South Africa	4	3,826	32
4	Manchikanti L	USA	4	18,434	78
5	Groot W	Netherlands	4	4,742	35
6	Friele R	Netherlands	4	1,652	21
7	Robben P	USA	4	338	10
8	Burnett SM	USA	4	369	9

**Figure 6:**
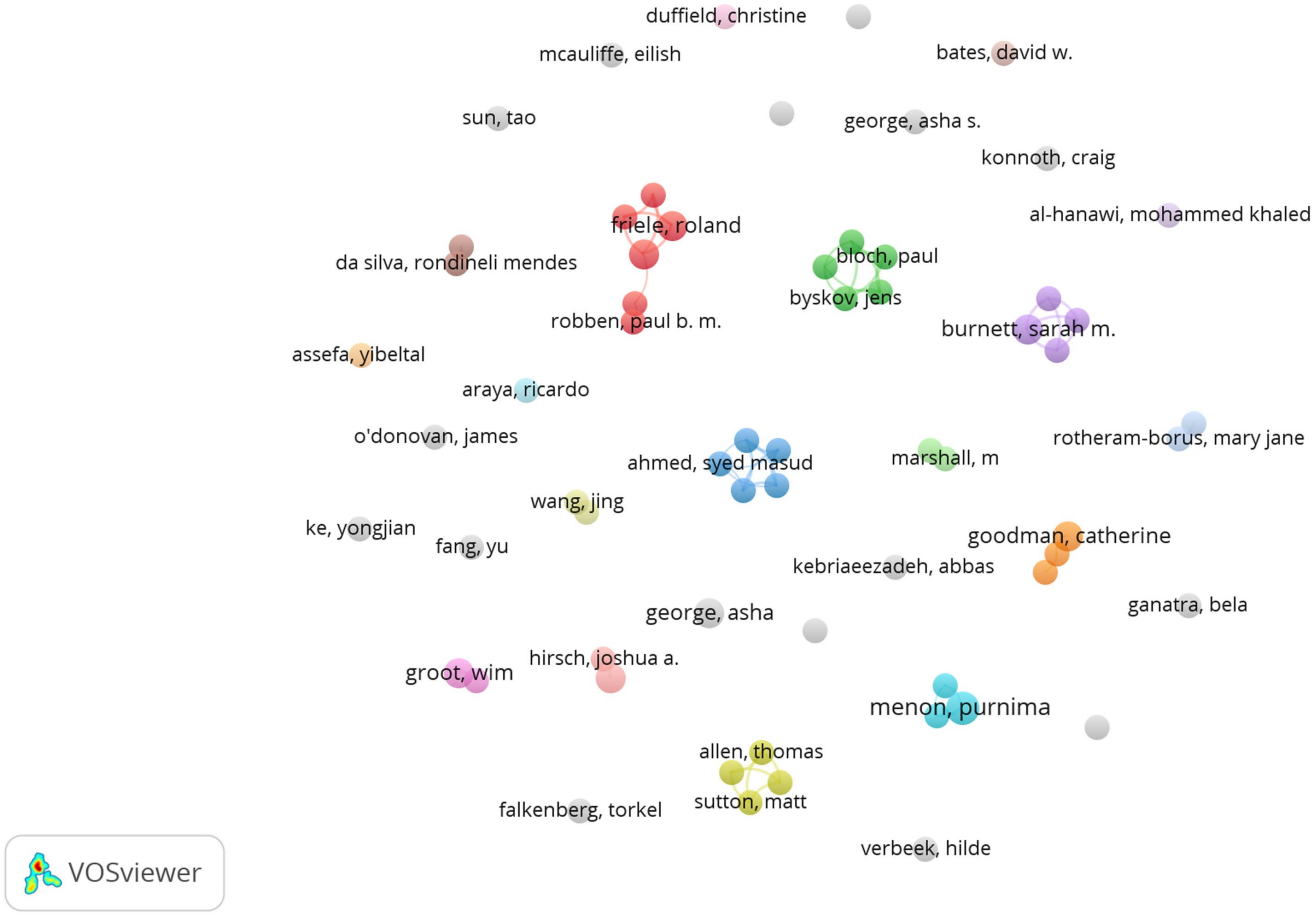
Collaboration network of authors.

Figure 6 shows the network visualization between the co-authors of GR study in healthcare. The size of a single node represents the number of publications of the author. Each color represents a different cluster. The biggest cluster mainly consisted of Bomhoff M, Bouwman R, Friele R, Robben P, and Stoopendaal A, who are from Netherlands. The next two largest clusters are “Bloch P, Byskov J, Hansen K S, Magnussen P, Mubyazi G M” and “Ahmed S M, Islam M A, Nizame F A, Rousham E K, Unicomb L, followed by the third cluster with the team of Allen T, Proudlove N, Sutton M, Walshe K and the team of Alombah F, Burnett S M, Hamilton P, Wun J.

### Journal analysis

A total of 1,830 papers were published in 976 academic journals. Table 2 presents the 10 most productive journals in the GR research. The most productive journal was the *BMC Health Services Research*, which has published a total of 70 articles since 2006, with the highest number of publications in 2022 (*n* = 12). Next is *Plos One* with 35 publications, with the highest number of publications in 2022. From the perspective of influencing factors in 2022, the highest is *Social Science & Medicine* (IF = 5.4), with a total of 29 publications published, and the earliest publication can be traced back to 1996. The next is *Frontiers in Public Health* (IF = 5.2), which publishes 27 articles since 2016, with the highest number of publications in 2023 (*n* = 10). In addition, the publications are mainly published in the journals of Q1 and Q2, which indicates the high quality of the publications to a certain extent.

**Table 2:** The top 10 most productive journals in GR research.

Rank	Journal	Count	IF2022^*^	Q^#^
1	BMC Health Services Research	70	2.8	Q3,Q2
2	Plos One	35	3.7	Q2, Q1
3	Health Policy	33	3.3	Q2,Q1
4	Social Science & Medicine	29	5.4	Q1
5	Health Policy and Planning	29	3.2	Q2
6	Frontiers in Public Health	27	5.2	Q1
7	BMC Public Health	26	4.5	Q2
8	International Journal of Environmental Research and Public Health	24	2.5^♦^	Q2,Q1
9	BMJ Open	19	2.9	Q2, Q1
10	International Journal of Health Planning and Management	18	2.7	Q2, Q3

^*^IF, Impar Factor; ^#^Q, Quartile in Category; ^♦^represents IF2018.

### Analysis of author keyword frequency

Word frequency analysis refers to a bibliometric method that analyzes research hotspots and development trends in a certain field by comparing the frequency of word occurrences in the literature (18). The keywords of the article reflect the core theme and main content of the publication, so this section conducts keyword frequency statistics on GR in healthcare.

Regulation, GR, healthcare, and their synonyms had a high frequency. But considering the use of these keywords in the search of this study, the analysis on them was of little significance, so they were excluded from the results. On this basis, the 15 keywords with the highest frequency were listed as shown in Table 3, and the frequency and total link strength were also listed. As shown in Table 3, COVID-19 is a research hotspot for GR in healthcare, due to its highest frequency and correlation strength. As formal tools of GR, policy, and law have been the focus of research, such as “health policy” and “legislation.” In addition, ethic, as an informal tool of GR, has also become the focus of research. In terms of methods, researchers tend to use qualitative research methods. Some scholars have also focused on government regulation of healthcare in China and India. Finally, “mental health,” “primary health care,” and “community health workers” have also become the focus of researchers.

**Table 3:** High frequency keywords for GR in healthcare.

No.	Keywords	Frequency	Total link strength
1	COVID-19	115	191
2	Health policy	83	164
3	Public health	60	127
4	Policy	44	73
5	China	40	46
6	Mental health	39	66
7	India	38	68
8	Primary health care	29	55
9	Qualitative research	28	60
10	Governance	27	52
11	Ethics	25	48
12	Pandemic	24	58
13	Legislation	23	47
14	Health systems	23	46
15	Community health workers	22	38

As mentioned above, the top 10 countries for GR research in healthcare are located in different regions. This section analyzes high-frequency keywords from different regions to examine the differences in government regulatory research priorities in healthcare. Table 4 presents the top 15 hot keywords with high frequency in different regions. As shown in Table 4, GR in healthcare in various regions mainly focuses on research on health policy, COVID-19, public health, mental health, and other aspects. Researchers in North America and Europe have focused on research on community health work, while in Asia, public-private partnerships have been studied. In addition, GR in India has been studied in North America and Europe. The European and Asian regions focus on using qualitative research methods. From a single regional perspective, GR research in North America focused on ethics, law, and telehealth. The European region focused on policy, governance, and primary health care, with research on government regulation in India and China. The Asian region focused on healthcare reform, rural health, and other areas of research.

**Table 4:** High frequency keywords in different regions.

No.	North America (USA/Canada)	Europe (UK/Netherlands/Germany/Switzerland)	Asia (China/India)
1	Health policy	COVID-19	China
2	COVID-19	Health policy	India
3	Public health	India	COVID-19
4	Policy	Public health	Health policy
5	Ethics	Governance	Mental health
6	India	Mental health	Public health
7	Mental health	Policy	Pandemic
8	Canada	Qualitative research	Healthcare reform
9	Public policy	Community health workers	Policy
10	Community health workers	Health systems	Rural health
11	Law	Primary health care	SARS-CoV-2
12	Pandemic	Quality	Systematic review
13	Health systems	Health	Qualitative research
14	Telehealth	Implementation	Public-private partnerships
15	Governance	China	Depression

Only involving regions that include two or more countries with the top 10 publications.

### Author keyword co-occurrence analysis

**Figure 7:**
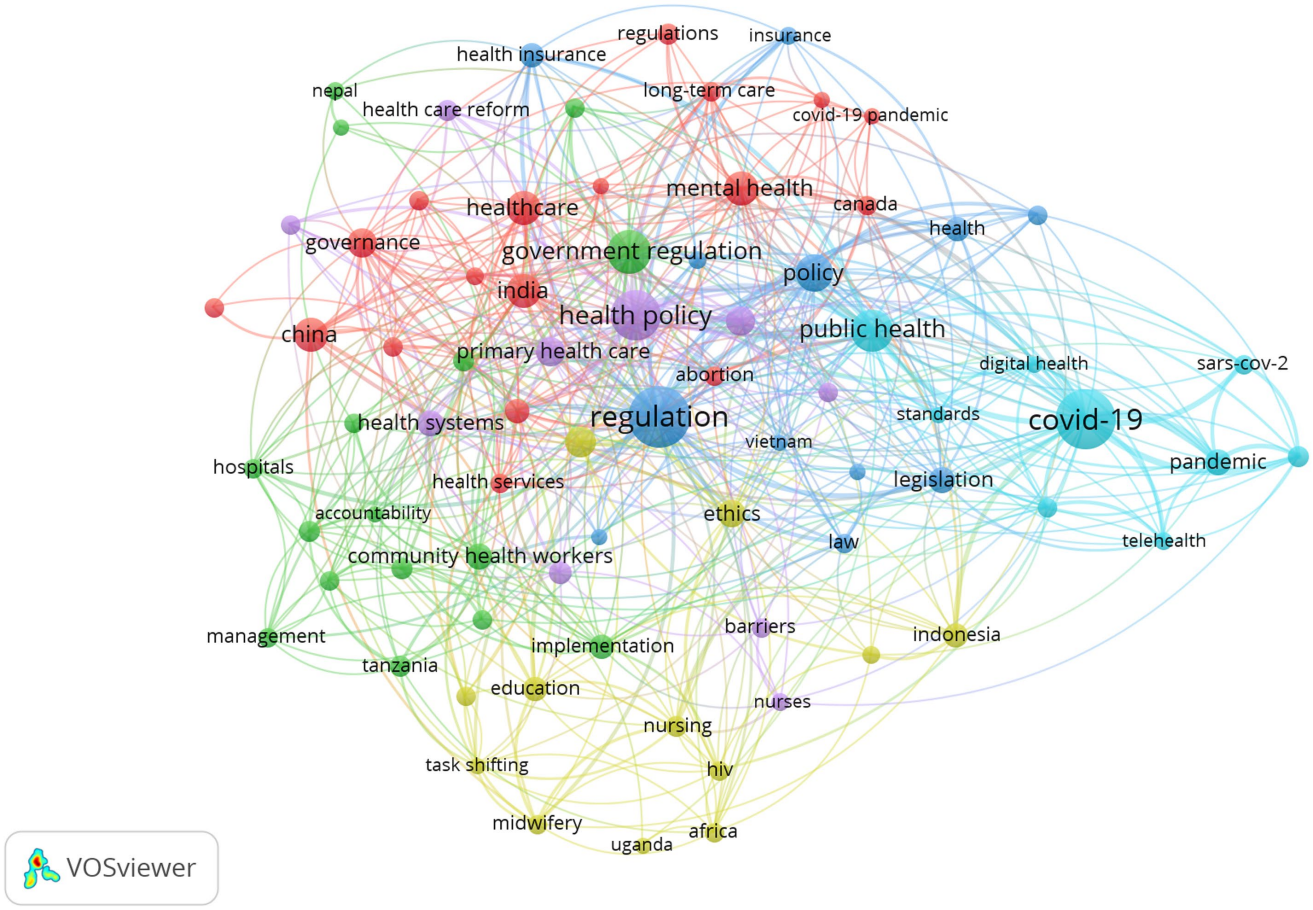
Keyword co-occurrence network.

This study draws a keyword co-occurrence network for GR literature in healthcare (Figure 7).

As can be seen in Figure 7, the government regulatory keyword co-occurrence network is deeply intertwined and complex, and different colors represent different clusters, with a total of 6 clusters that overlap more, making it difficult to directly see more specific information. Therefore, it is necessary to further explore the information contained in each cluster category, analyze the text information corresponding to each cluster, and statistically summarize the basic situation of each cluster category in the keyword co-occurrence network clustering graph, as shown in Table 5, reflecting current research hotspots.

**Table 5:** Keyword co-occurrence network clustering statistics of GR in healthcare.

Cluster number (color)	Cluster labels	Research focus	Items of clusters
I (red)	Healthcare	Mental health	18
		China	
		India	
		Governance	
II (green)	Government regulation	Community health workers	16
		Public policy	
		Patient safety	
		Quality	
III (blue)	Regulation	Policy	12
		Legislation	
		Health insurance	
		Law	
IV (yellow)	Health care	Ethics	12
		Education	
V (purple)	Health policy	Qualitative research	10
		Primary health care	
		Health care reform	
VI (baby blue)	COVID-19	Public health	9
		Telemedicine	
		Digital health	
		Pandemic	

**Figure 8:**
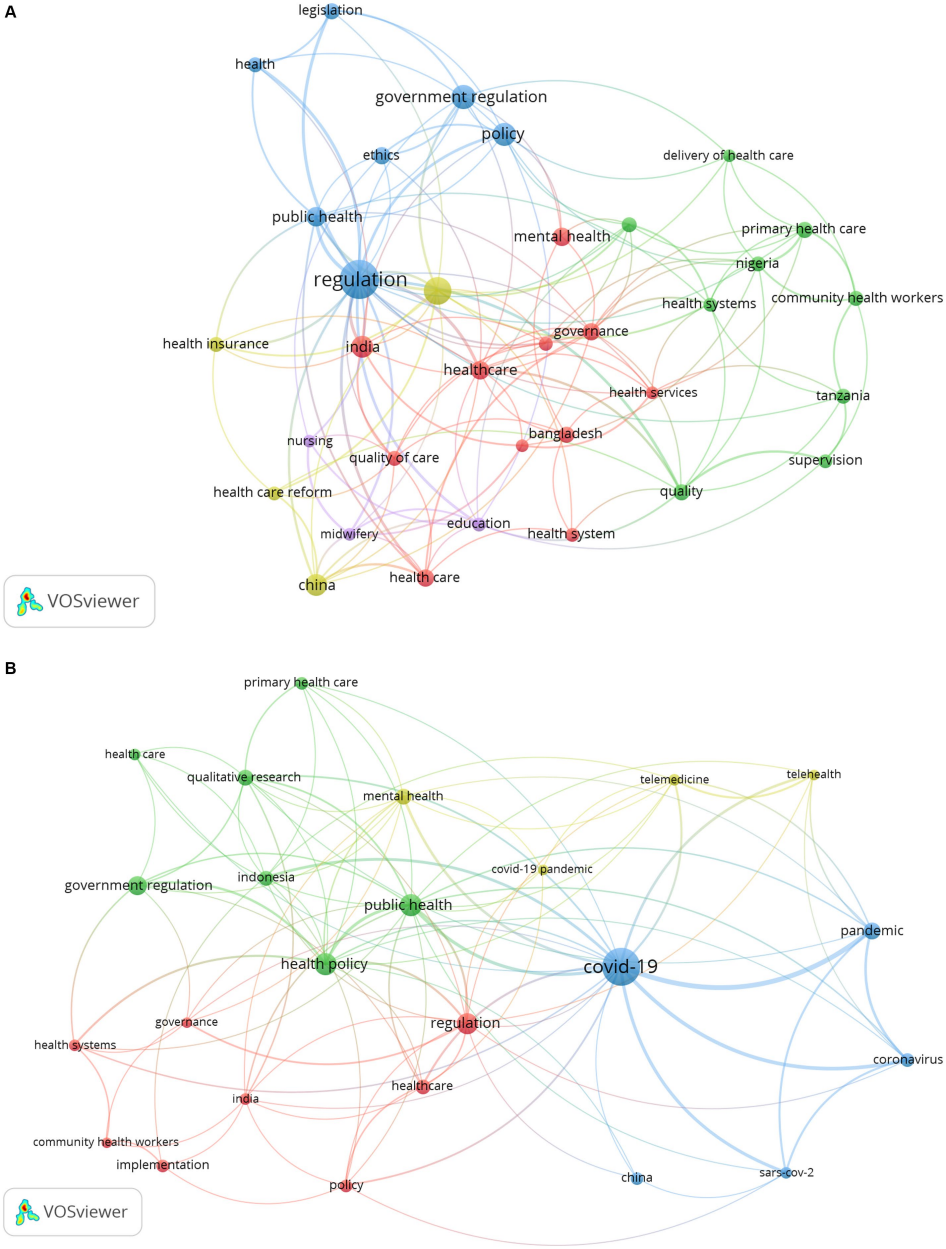
Keyword co-occurrence network: **(A)** before the outbreak of COVID-19; **(B)** after the outbreak of COVID-19.

To further analyze the differences in government regulatory research in healthcare before and after COVID-19, we divided the literature retrieval into two stages: 1996–2019^1^ and 2020–2023. And the Vosviewer software was used to conduct keyword cluster analysis of the literature in these two stages (Figures 8A,B). As shown in Figure 8A, before COVID-19, GR research in healthcare focused on mental health, legislation, quality, primary health care, community health workers, and other aspects. In particular, some studies have been conducted on GR in healthcare in India (quality of care, equity, governance, etc.), China (health care reform and health insurance, etc.), and Bangladesh (health services, etc.). Figure 8B presents topics related to GR research in healthcare after COVID-19 outbreak. It can be seen that after COVID-19 outbreak, research on the virus has become a hot topic in GR research in healthcare. And words such as “pandemic” and “coronavirus” have become high-frequency keywords. In response to COVID-19, the application of telemedicine has been significantly improved, becoming a research focus after COVID-19. In addition, after COVID-19, qualitative research has become an important research method for researchers.

## Discussion

Within the aim of the research, the current bibliometric analysis provided information on the structure of GR publications in the healthcare field, which helped researchers identify publishing activities related to journals, keywords, authors, etc. Since 1996, the number of GR academic publications has steadily increased in fluctuations, reaching a peak in 2021 as of July 2023. The earliest record was in 1996 and consisted of four articles. One discussed the impact of regulations, accreditation standards, and healthcare reform on laboratory operations in the United States (19), and the other discussed Sweden’s drug reimbursement system in the context of rising public drug spending, pointing out the need for the government to improve regulation of this system, thereby improving its efficiency and controlling costs (20). The other two papers are from the UK and mainly explore policies related to the development of private healthcare (21) and the regulation of drug prices in the pharmaceutical market (22). In addition, most of the articles appeared after 2017, especially in 2020–2022, which has great relevance to the COVID-19.

### Discussion on countries and institutions

Both developed and developing countries conducted research on GR. There are more publications in developed countries, and the research time is earlier. But this result may be related to considering only English publications. In recent years, the amount of research in developing countries has also been increasing, especially during the global COVID-19 pandemic in 2019, which pushed the research related to GR in healthcare to a peak. However, the number of articles published by developed countries is 4 times that of developing countries, accounting for 76.8% of the total number of articles. In our study, a large number of GR publications were published in North America (e.g., USA, Canada), Europe (e.g., UK, Netherlands, Switzerland, Germany), and Asia (e.g., China, India). Among them, the United States is the most productive country that has conducted GR research since 1996, followed by the United Kingdom since 1996. South Africa and India have conducted research on GR since 2003. In addition, Canada, the Netherlands, and Germany have conducted studies on GR in healthcare since 2000. In general, both developed and developing countries have made certain contributions to GR research. Correspondingly, institutions in these countries, such as the University of London and London School of Hygienetropical Medicine in the United Kingdom, Harvard University, Johns Hopkins University, and University of California System in the United States, and the University of Toronto in Canada, have also produced more results.

### Discussion on author collaboration network

In the author’s collaboration network, there are five teams in the current research field. The first is a research team composed of Bomhoff M, Bouwman R, Friele R, Robben P from the Netherlands. The team has been focusing on GR research since 2015, mainly focusing on the perspectives of patients and regulatory agencies on a certain issue. For example, they argue that patients and regulatory agencies are committed to improving health-care quality, but patients’ perceptions of the complaints’ relevance differ from the regulator’s perceptions (23). They also conducted research on the perspectives of patients and Regulators on healthcare quality (24), as well as public’s voice about healthcare quality regulation policies (25). In addition, before 2013, the author with the most cooperation with Friele R was Coppen R from the Netherlands Institute for Health Services Research, who mainly engaged in research on Organ donation and other aspects. Second, Mubyazi GM, Bloch P, Byskov J, Hansen KS, Magnussen P and others collaborated closely and jointly published 3 articles, focusing on exploring malaria prevention research during pregnancy in women in Tanzania (26, 27). The third team is Islam MA, Ahmed SM, Nizame FA, Rousham EK, and Unicomb L, from the UK, Bangladesh and the US. They collaborated to study the driving factors of antibiotic use in Bangladesh to translate into policy development and implementation (28). The fourth research team, Allen T, Proudlove N, Sutton M, and Walshe K from the University of Manchester in the United Kingdom, collaborated on a study of the indicator system designed and used by regulators and suggested that assessing predictive power should be undertaken prospectively when the sets of indicators were being designed and selected by regulators (29, 30). The fifth team, including Alombah F, Burnett S M, Hamilton P, and Wu J, collaborated on research on Malaria Case Management Supportive Supervision (31, 32).

### Discussion on author keyword frequency

Keyword analysis of publications not only provides an effective method for studying the knowledge structure of the field, but also provides an effective way to explore the development trends in the field (33). From the perspective of high-frequency keywords (Policies, Health policies), policy tools have always been an important research object for the government to maintain public health in healthcare, including, etc. For example, price control policy (expenditure cap) (34), drug pricing policy (35–37), anti-monopoly regulatory policy (38–41), doctor’s dual career policy (42), and digital health policy derived from the application of digital technology in healthcare (43).

High-frequency keywords also reflect that the GR in healthcare is still command-and-control, relying on the mandatory law to supervise health care-related behaviors. Mello conducted a study on the role of law in public health and proposed that legal rules affect information people receive about family planning through channels such as pre-marital counseling and the mass media. They affect the range of contraceptive products available, for example, restrictive local ordinances may eliminate access to prohibited products, while prescribing rules may make contraceptives more or less difficult to obtain. They also affect the affordability of contraceptives by regulating (or not regulating) prices and imports, imposing taxes and tariffs, and setting public insurance coverage rules (44). The Health Insurance Portability and Accountability Act of 1996 provides legal support for telemental health development as a minimum standard to protect the privacy of patients when their health information is transmitted electronically to business partners, such as insurance providers. And the Health Information Technology for Economic Clinical Health Act of 2009 adopted by HIPAA and DHHS in 2010 further strengthened the security rules for the transmission of patient health information (45). Furthermore, from within the government, there is a lack of consistency between laws at the central (national) level and legislation at the local government level. For example, in Spain, even though no specific national law has as yet been passed to regulate medical waste management, 13 regional governments have adopted regulations to protect health (46). At the horizontal level, different state and local governments have different regulatory rules. For example, during the COVID-19 pandemic, states in the United States have adopted different policies in healthcare, mainly including telemedicine, privacy, and medications for opioid use disorder (47).

Due to the professionalism and complexity of medical knowledge, the regulatory approach of negotiation and cooperation has gradually become an important means of regulation. The government encourages medical associations and non-governmental organizations to participate in the establishment of relevant guidelines and standards in the medical field, making them an important supplement to GR. For example, the standards and guidelines (e.g. the Guidelines on Gifts to Physicians from Industry) developed by the American Medical Association play an important role in regulating medical behavior, and are recognized by the government. Federal law enforcement officials consider compliance with voluntary codes and standards issued by medical societies and industry as the minimum standard for complying with federal and state anti-kickback laws (48). Bork et al. proposed that successful public health system reform requires restructuring the functions and influence of stakeholders in the healthcare system, and constructing a comprehensive healthcare governance framework that includes independent third-party supervision (49).

From the perspective of high-frequency keywords in different regions, the research on GR in healthcare in India has become a focus of research in North America and Europe, which to some extent indicates a cooperative relationship between the North America and Europe regions and India in academic research on GR in healthcare. For example, authors Vallath and Tandon from India, Pastrana from Germany, and Lohman and Husain from the United States collaborated on research on drug policy reform in India (50). Peters, from Johns Hopkins Univ, Bloomberg Sch Publ Hlth, USA, and Rao, from Andhra Medical College, India, worked together to study health policy in India (51). In addition, China, as a high-frequency keyword, has also become the focus of GR research in healthcare in the European region, indicating that the country in Europe has cooperated with China in academic research and exchange. Wang from Zhejiang University in China, Wu from the London School of Hygiene & Tropical Medicine in the UK, and Xuan from Boston University in the United States collaborated to study the impact of the Chinese government’s policy of banning outpatient intravenous antibiotic treatment on secondary and tertiary hospitals in China (52). In general, the cooperation and exchange of academic research in different countries can help broaden the depth and breadth of GR research in healthcare.

### Discussion on changes in GR before and after the outbreak of COVID-19

According to the keyword cluster analysis before and after the outbreak of COVID-19, COVID-19 has brought major challenges to the global healthcare system (53), resulting in some changes in GR research in healthcare, which are reflected in the following three aspects.

Firstly, in terms of the number of publications, after the COVID-19, the number of publications on GR in healthcare increased significantly. Only in the 3 years of 2020, 2021, and 2022, 628 publications were published, accounting for 34.3%, with an average of 209.3 published annually. Among them, the largest increase in the number of publications was 55 in 2020. From the perspective of keyword analysis of these 3 years, these studies mainly focus on COVID-19.

Secondly, the application of telemedicine. The COVID-19 pandemic has required healthcare systems to radically and rapidly rethink the delivery of care, and one of the most significant changes is the unprecedented acceleration of telehealth expansion (54). According to the 2019 Health Center Project Data in the United States, 43 percent of healthcare centers were able to offer telemedicine, compared to 95 percent of healthcare centers that reported using telemedicine during the COVID-19 pandemic (55). This rapid development is due not only to the unique advantages of telemedicine itself to meet people’s needs for healthcare during COVID-19, but also to the relaxation of government regulations on telemedicine to promote the development of telemedicine during the epidemic. For example, the federal government in the United States relaxed regulations on telemedicine, effectively removing some of the biggest regulatory barriers that had limited the adoption of telehealth (56). In addition, the original Clinical Laboratory Improvement Amendment regulation required pathologists to electronically verify patient reports from certified institutions. However, during the COVID-19 pandemic, the government relaxed the enforcement of this regulation, allowing pathologists to review and report pathological specimens from remote non-CLIA certified facilities (57). The illegal status of telemedicine was lifted to follow established patients through telemedicine in South Korea (7). However, the use of telehealth in emergency situations has led to a variety of different practices, different definitions of what constitutes telehealth or telemedicine, and different rules, which not only lead to difficulties in designing and implementing telehealth research across states, but also to confusion between patients and healthcare providers, and makes it difficult to implement policies that are needed across states or on a national level (58).

Thirdly, keyword analysis found that in terms of research methods, GR researchers tend to use qualitative research methods, especially scholars from Europe and Asia. This is because qualitative research can help researchers gain a deeper understanding of stakeholders’ perspectives, ensuring that their perspectives and experiences are captured when evaluating the consequences of policy implementation or providing information for regulatory design. Whether reviewing the consequences of regulations on stakeholders or bringing their voices into regulations developed around health issues—patient safety, qualification, competence, scope of practice, etc.—qualitative research can provide exploratory and interpretative data (59). Blackman also pointed out that qualitative research plays an important role in ensuring that stakeholders’ voices are represented and their experiences inform the evaluation of regulations and related policies when studying nursing and other health professions (60). For example, Radevic used a case study method to analyze Montenegrin legislation and obtained relevant data through individual and group interviews with top executives in the Ministry of Health of Montenegro, the Health Insurance Fund of Montenegro, and the Ministry of Public Administration (61). Oyri conducted a case study on the implementation and impact of the Quality Improvement Regulations, conducting three focus groups and two individual interviews with regulatory inspectors to explore whether and how regulatory methods in Norwegian hospitals have changed (62). In addition, Nxumalalo used a comparative case study method to compare and analyze three CHW projects providing healthcare services in two provinces, namely the Eastern Cape and Gauteng in South Africa (63).

### Limitations of the study

Our research studied the global trends and application status of GR research in the domain of healthcare over 28 years from the WoSCC database. It enhanced the scientific nature of research that will help generate evidence-based descriptions, comparisons, and visualizations of research findings in GR through the use of Vosviewer and Excel. Nevertheless, there are some limitations to our analysis. First, the publication search is only conducted in the WoSCC database and only in English, which may lead to selection bias due to some research omissions. Second, we only analyzed the country distribution, authors, keywords, and other information of the research results from the perspective of bibliometrics, without a specific analysis of the research content. Therefore, this analysis may not provide a better overview of the content of the literature on GR.

## Conclusion

This article studied the current research status and application of GR in the field of healthcare based on publications from the WoSCC database. Over the past few decades, scholars have been studying GR in the field of healthcare. But in the last 3 years, the outbreak of COVID-19 has led to a significant increase in the number of studies on GR in the field of healthcare, making it a key focus for researchers. From the perspective of country distribution, although the research results on GR in developing countries were constantly improving, most research results were still concentrated in developed countries. The author teams mainly come from the combination of Bomhoff M, Bouwman R, Friele R, Robben P, as well as the group of Mubyazi GM, Bloch P, Byskov J, Hansen KS, and Magnussen P. From the perspective of high-frequency keywords, policy tools (such as health policy) have always been an important tool for GR. Law and Legislation, as an important tool for mandatory regulation, have still received attention and become a research hotspot. The professionalism and complexity of the medical field make negotiated regulation necessary, forming a multi-body governance structure consisting of medical industry associations, other non-governmental organizations, and community service workers. Correspondingly, medical industry standards, guidelines, and ethical guidelines serve as informal regulatory tools to guide and constrain medical behavior. These are in accordance with that keywords such as ethics and governance become research hotspots. In addition, mental health has always been a focus of GR research in healthcare. GR in healthcare in India has become a research focus in North America and Europe, while GR in healthcare in China has also received attention from some European researchers. This also indirectly indicates that scholars from these two countries have conducted more academic cooperation and exchanges with some European and American countries in the research of GR in healthcare. In addition, in terms of research methods, many researchers have used qualitative research methods to study GR in healthcare, especially in European and Asian. Finally, the keyword cluster analysis found that research on GR in healthcare has changed after COVID-19. First, the number of publications on GR in healthcare has increased significantly, making keywords such as COVID-19 and pandemic become research hotspots; Second, telemedicine has developed rapidly; Third, qualitative research methods have been widely used. With the end of COVID-19, further research is needed on how to handle policies, laws, etc. that generated during the COVID-19 period, whether to retain or abolish them. In addition, with the continuous development of information technology, telemedicine will be increasingly applied, so future researchers need to conduct more systematic and specialized research on telemedicine. Finally, it is hoped that our research findings can provide some useful information for GR in healthcare, and provide some reference for policy makers and some researchers.

## Author contributions

MQ: Conceptualization, Formal analysis, Methodology, Writing – review & editing, Data curation, Investigation, Project administration, Resources, Software, Visualization, Writing – original draft. JR: Conceptualization, Formal Analysis, Methodology, Writing – review & editing, Supervision, Validation.

## Footnotes

^1^Since COVID-19 broke out at the end of 2019, and academic research on practical problems has a certain time lag, we attribute the relevant research in 2019 as “before COVID-19.”
